# Obesity and myeloma: Clinical and mechanistic contributions to disease progression

**DOI:** 10.3389/fendo.2023.1118691

**Published:** 2023-02-23

**Authors:** Constance Marques-Mourlet, Reagan Di Iorio, Heather Fairfield, Michaela R. Reagan

**Affiliations:** ^1^ MaineHealth Institute for Research, Center for Molecular Medicine, Scarborough, ME, United States; ^2^ University of Strasbourg, Pharmacology Department, Strasbourg, France; ^3^ University of New England, College of Osteopathic Medicine, Biddeford, ME, United States; ^4^ University of Maine, Graduate School of Biomedical Science and Engineering, Orono, ME, United States; ^5^ Tufts University, School of Medicine, Boston, MA, United States

**Keywords:** obesity, multiple myeloma, diet, treatment, hematological malignancies

## Abstract

Obesity and obesogenic behaviors are positively associated with both monoclonal gammopathy of unknown significance (MGUS) and multiple myeloma (MM). As the only known modifiable risk factor, this association has emerged as a new potential target for MM prevention, but little is known about the mechanistic relationship of body weight with MM progression. Here we summarize epidemiological correlations between weight, body composition, and the various stages of myeloma disease progression and treatments, as well as the current understanding of the molecular contributions of obesity-induced changes in myeloma cell phenotype and signaling. Finally, we outline groundwork for the future characterization of the relationship between body weight patterns, the bone marrow microenvironment, and MM pathogenesis in animal models, which have the potential to impact our understanding of disease pathogenesis and inform MM prevention messages.

## 1 Introduction

Multiple Myeloma (MM) is a fatal plasma cell dyscrasia characterized by bone marrow (BM) infiltration and lytic bone lesions. MM is the second most common hematologic malignancy, with approximately 34,460 new cases diagnosed in 2022 in the USA alone (1). Advancements in MM treatments have dramatically improved the median survival time in patients over the past two decades; however, MM remains incurable. Existing evidence points to obesity or obesogenic behaviors as plausible targets for MM prevention. Obesity remains one of the few established risk factors for both MM and its premalignant precursor states of monoclonal gammopathy of unknown significance (MGUS) and smoldering MM (SMM), and is the only known modifiable risk factor. For individuals at risk for MM, further understanding of the relationship between disease risk and body weight are urgently needed.

Obesity, defined as a body mass index (BMI) ≥30, affects approximately 42% of adults in the US (≥18 years of age), and has been rising concurrently with the prevalence of cancer over the past two decades (2). Obesity-related conditions (eg. heart disease, stroke, type 2 diabetes and certain types of cancer) led to medical costs of ~$173 billion in 2019, and these conditions are among the leading causes of preventable, premature death in the US (2). Extremely high BMI has been associated with an increased risk of at least 13 cancers, including MM (3–6), and many studies have demonstrated a significant reduction in cancer risk with weight loss in women (7). Adipose tissue, commonly known for its role in energy storage, commonly increases with weight gain and increased BMI, and represents an endocrine organ that secretes bioactive compounds. It is composed of adipocytes, macrophages, lymphocytes, preadipocytes, fibroblasts, and endothelial cells. Adipose tissue composition depends on type, location, age, and degree of obesity, among other factors. Two types of adipose tissue most well studied are white adipose tissue (WAT) and brown adipose tissue (BAT) (8), but recent work has begun to characterize other adipose depots such as bone marrow adipose tissue (BMAT) (9), beige adipocytes (10, 11) and perivascular adipose tissue (PVAT) (12). The contributions of these depots to obesity/metabolic disease, and their responses to obesity, may suggest new anti-obesity treatments, or further illuminate how obesity initiates such devastating diseases. Specific to MM, most research currently investigates contributions of WAT or BMAT to MM, since the BM is the primary location of myeloma cell growth, and since both WAT and BMAT increase in obesity (13). As myeloma cells are known to interact closely with neighboring cells, their interaction with BM adipocytes represents an interesting new field of research, especially since BMAT also increases with age (14), another major risk factor for MM.

Some studies have attempted to understand the link between increased MM risk and obesity through biological mechanisms, as described below. Adipokines, such as leptin and IL-6, induce myeloma cell survival and proliferation, but targeting adipokines in MM or cancer generally has not had great translational success and new targets, based on a better understanding of the pathology, are needed. Very few studies specifically interrogate the mechanistic links between obesity and myeloma disease progression, perhaps due to technical challenges of preclinical models, and thus our overall understanding of the effects of obesity on MM and its microenvironment, is still nebulous. The aim of this review is to provide a summary of the current epidemiologic evidence associating obesity with myeloma incidence, the status of the field attempting to connect cellular mechanisms to this increased risk, and our recommendations for preclinical (animal) studies to address some of the remaining gaps in this field.

## 2 Obesity, diabetes, and multiple myeloma

### 2.1 Obesity and risk for MM incidence

Obesity has long been considered a major risk factor for cancer and is the only known modifiable risk factor for multiple myeloma. At every stage of the disease progression, obesity plays a part, but the greatest impact may happen before a diagnosis is made, in the precursor disease state. Monoclonal gammopathy of undetermined significance (MGUS) is a premalignant condition defined by the presence of a monoclonal paraprotein in the blood, a proliferation of clonal plasma cells in the BM (less than 10%), and the absence of end organ damage (15). MGUS occurs in 3.2% of persons aged 50 and older and 5.3% of those over age 70 (16), and it has a persistent risk of progression to MM of about ~1% per year (17). A recent systemic review found that increased BMI and obesity are implicated in MGUS development, as well as the progression to overt MM (18). Many other studies agree: the International Agency for Research into Cancer (IARC) and the World Cancer Research Fund cite greater body fatness as a contributor to the risk for MM development (5). The risk ratio (RR) of myeloma incidence has been found to be significantly increased (RR=1.11, p<0.0001) in both women and men, analyzed separately, based on a meta-analysis of 6 or 7 studies, respectively (per 5 kg/m^2^ increase in body weight) in a 2008 study (18). A retrospective study of PET/CT data found that patients who were recently diagnosed with MM had higher abdominal fat cross sectional area and higher fat metabolic activity compared to patients with MGUS (19).

The Age Gene/Environment Susceptibility-Reykjavik Study (AGES-RS) has since revealed that a high midlife BMI is associated with increased risk of progression from MGUS to MM and other lymphoproliferative diseases (20). Similarly, a retrospective study from the US Veterans Health Administration in 2017 showed weight status and obesity are both associated with increased risk of transformation of MGUS to MM (21). Most recently, a retrospective analysis in 2022 within the Prostate, Lung, Colorectal and Ovarian (PLCO) Cancer Screening Trial revealed a 35% increase in odds of progression from non-IgM MGUS to MM with each 5 kg/m^2^ increase in BMI (22).

In 2018, data gathered from the Nurses’ Health Study (NHS), Health Professionals Follow-Up Study (HPFS), and Women’s Health Study (WHS), revealed a positive association of both cumulative average adult BMI and young adult BMI with MM risk (23). MM incidence was positively associated with adult BMI, with relative risks of 1.2 for overweight, 1.2 for class 1 obesity, and 1.5 for class 2 or 3 obesity (6). Over a twenty-year observational period, MM risk is increased with increased hip circumference and medium-increase body trajectory compared to lean-stable body trajectory. Interestingly, extreme weight cycling (with net weight gain and at least one episode of 20 pound or greater weight loss) was recently associated with increased risk for MM compared to maintained weight (24).

### 2.2 Obesity is a controversial factor in multiple myeloma progression and MM-induced mortality

A prospective study on the influence of excess body weight and risk of death from cancer found current patterns of obesity in the United States could account for 14% of all deaths from cancer in men and 20% in women (4). The relative risk of death from cancer for men was 1.52 and 1.62 for women, with BMI significantly associated with death due to MM specifically (4). Pooled analyses of prospective trials from 2011 and 2014 revealed a 9-15% and 52-54% increased risk of MM mortality for overweight (BMI ≥25) and obese individuals, respectively (25, 26). In African American (AA) populations, mortality due to MM increases monotonically with BMI increase, with hazard ratios up to 1.43 for BMIs of 35 kg/m^2^ or greater, using data from seven prospective cohorts tracking mortality among 239,597 adults in the AA BMI-Mortality Pooling Project (27).

Still, not all studies agree, and a recent study of 563 subjects showed BMI before (for both sexes) and at the time of MM diagnosis (for males) are not associated with overall survival in MM, and, surprisingly, that a higher BMI at diagnosis is associated with *better* overall survival for females (28). Similarly, one abstract presented at the 16^th^ International Myeloma Workshop in 2017 by a team from the Mayo Clinic found that, in patients with heavily pretreated MM, obesity (BMI >30) was associated with better outcomes compared to those who were not obese (BMI <30) (29). Interestingly, although the response rates for these groups to treatments were similar for the obese and non-obese groups, the progression free survival (PFS) and overall survival (OS) were significantly better in the obese group (29). The authors explain the that exact cause of this is unknown, but hope that future work will both confirm their findings and explore the mechanisms that physiological consequences of obesity may have on disease biology or drug metabolism (29).

Similarly, in a study of 1,087 MM patients, among those who received melphalan and total body irradiation (TBI), obese and severely obese patients had superior PFS and OS than did normal and overweight patients: PFS at 5 years was 23% in normal weight patients, 17% in overweight patients, 43% in obese patients, and 55% in severely obese patients (p=0.005) (30). This was not true for MM patients who received melphalan alone, suggesting that differences in how irradiation affects MM in obese vs lean individuals may drive the differences in patient responses (30). Overall, some studies support an associate between increased BMI or obesity, and increased mortality in MM, while other studies suggest that obesity can, surprisingly, have the opposite effect on MM patient outcomes. Thus, more research into obesity and response to MM treatments is needed and more work should be done to determine if weight loss strategies (diet, exercise, or drugs) are useful interventions in MM.

The controversial finding that obesity, a major risk factor for many cancers, can be protective in terms of cancer patient survival has been observed in many other types of cancer as well, and is termed the “obesity paradox” (31). As reviewed by Arnold et al., there are perhaps biological explanations for this, such as protection from cachexia [which occurs in ~30% of MM or lymphoma patients during treatment and increases mortality risk (32)], or advantages of increased body weight during aging. However, there are also a many plausible methodological reasons for the “obesity paradox”, such as residual confounding, reverse causality, and a selection bias known as “collider bias” (31). In fact, blocking browning in white adipose seems to be an effective way to protect from cachexia-induced weight loss and muscle wasting, and thus it is likely not the total amount of adipose, but it’s metabolic state, that determines its role in cachexia (33, 34). Furthermore, cachexia, a metabolic syndrome that can be present even in the absence of weight loss, can in fact frequently be obscured by obesity, leading to under-diagnosis and excess mortality (35). Thus, we should not interpret the observations about obesity and survival in MM, or any cancer, to mean that high BMI will reduce the risk of death or cachexia for cancer patients, or that gaining weight could be beneficial (31).

### 2.3 The diabetes- MM connection

Many publications of epidemiological data have found or suggested that type 2 diabetes mellitus (T2DM) is a risk factor for developing MM or causing worse clinical outcomes (36). For example, recent work from Shah et al. found that patients with MGUS, MM, amyloidosis, as well as some other lymphoproliferative disorders (LPDs), were more likely to have a preceding diabetes mellitus (DM) diagnosis compared to matched controls (37). However, the story is complicated, as they found that patients with DM were no more likely to progress from MGUS to MM, WM, amyloidosis, or other LPDs than non-diabetic patients. However, they note that they could not control for the use of anti-diabetic drugs that may lower rates of MGUS progression (37). A recent systematic review and meta-analysis analyzed 13 studies and concluded that T2DM does not increase risk of MM (34), but again the authors hypothesize that the use of hypoglycemic drugs, such as metformin, could explain why these patients do not have an increased risk of MM (34). Another recent study from Japan of 131,701 cancer patients, 6,135 of which had coexisting diabetes, found, perhaps not surprisingly, that survival was better for cancer patients without (versus with) diabetes (38). More interestingly, patients who had diabetes also had a higher risk of developing second primary cancer, specifically of MM, as well as uterine, liver, and pancreatic cancer (38). The presence of diabetes was identified from the prescription records of antidiabetic drugs, which included an array of drugs (metformin, insulin and insulin analogs (pen-type injection device), glucagon-like peptide 1 receptor agonists, dipeptidyl peptidase-4 inhibitors, sodium–glucose cotransporter 2 inhibitors, thiazolidines, glinides, alpha-glucosidase inhibitors and sulfonylureas) (38). Thus, even for patients on anti-diabetic drugs, diabetes was a risk factor for MM development (as a secondary cancer) (38). More on diabetes and MM specifically can be found in the reviews by Tentolouris et al. (38) and Fais et al. (39).

## 3 Treatment options for targeting obesity-related contributions to MM

The association between obesity and MM prompts a compelling argument for the use of metformin as a pharmacotherapy in patients with MGUS to reduce body weight, metabolic disease, or adiposity parameters, including BMI and waist circumference (40). Relatedly, metformin, a drug commonly used in diabetic patients, has promising anti-MM effects based on epidemiological data. Data from 2017 show a significant reduction in myeloma risk for patients with MGUS and cumulative metformin exposure >2 years and adjusted for the serum glucose level (41). Likewise, a cohort of US veterans with comorbid MGUS and diabetes mellitus (DM) treated with metformin displayed reduction in myeloma risk with metformin exposure >4 years (21). Similarly, in a recently study of 739,553 patients from Taiwan’s National Health Insurance database, T2DM patients who were prescribed metformin within the first year of their diagnosis with T2DM had a lower risk of developing MM compared to T2DM patients who were not prescribed metformin (42).

Metformin also exhibits anti-myeloma effects *in vitro* and immunocompromised xenograft models (43), however this has not been tested in a diet-induced obesity (DIO) model. A recent study on *in vitro* and *in vivo* MM models demonstrates decreased proliferation of dexamethasone-resistant and -sensitive MM cell lines treated with metformin, with cell cycle assays showing arrest in the G0/G1 phase of MM.1S and H929 cells and arrest in the G2/M phase of RPMI8226 cells (44). Mouse survival was prolonged with metformin treatment, with decreased U266 and H929 growth in BM (44). Moreover, metformin can reverse some of the negative effects on bone cause by DIO in mice (45), and has been shown to be beneficial to bone in other *in vivo* settings (46), which could make it useful in slowing osteolytic disease in MM. However, not all data on metformin concur; a recent study found mice pretreated with metformin for 4 weeks prior to inoculation of 5TGM1 MM cells had increased tumor burden, associated with increased osteolytic bone lesions and elevated osteopontin (OPN) expression in the bone marrow (47). *In vitro*, metformin increased MM cell attachment to osteoblasts, and increased OPN expression in preosteoblasts. This unexpected indirect pro-tumorigenic effect of metformin highlights the importance of fully elucidating the effects of metformin before using it as a treatment in MM (47). The implications of whether metformin could be repurposed in either MGUS as a preventative measure or in myeloma patients requires further investigation, as do other metabolically-focused therapies (40). A clinical trial is ongoing to assess whether metformin could be used in the future to help prevent MGUS or smoldering MM patients from progressing to MM (https://clinicaltrials.gov/ct2/show/NCT04850846).

Statins, or HMG-CoA reductase inhibitors, are another class of lipid-lowering medications that hold promise in MM (48). An analysis of 5,922 patients diagnosed with MM within the study period, the use of statins was associated with 21% reduction in risk of death among all patients, and of only those patients treated with novel agents (n=3,603), statins reduced mortality by 10% (48).

There is also promise in lowering MM risk through lifestyle changes promoting lower BMI. Data from the Women’s Health Initiative Observational Study (WHI-OS) demonstrates significantly lower obesity-related cancer risk in women with intentional weight loss (greater than 5%) over three years compared to women with stable weight. These results are independent of race and/or ethnicity, baseline BMI, smoking status, or prior hormone use, illustrating the modifiable nature of obesity as a risk factor (7). Likewise, a retrospective study from 2008-2017 revealed decreased incidence of obesity-related cancer in patients with comorbid nonalcoholic fatty liver disease (NAFLD) and obesity who underwent bariatric surgery compared to patients with NAFLD and obesity who did not (49). Furthermore, high leisure-time physical activity is associated with a 7% lower risk of obesity-related cancer compared to low physical activity, and when combined with a BMI <25, the relative risk reduction is 27% for MM (50). Bariatric surgery would likely be beneficial for patients with morbid obesity, as lower overall cancer risk has been seen in obese women within the first 5 years after bariatric surgery (51), and based on the growing body of evidence to support the role of obesity as a dynamic influence on MM incidence. However, the fact that bariatric surgery, such as vertical sleeve gastrectomy and Roux-en-Y gastric bypass, cause bone loss (decreased bone mineral density), increased bone turnover, and increased risk of fracture cannot be overlooked for MM patients who already face bone loss and high fracture risk (52, 53). Overall, evidence for weight loss as an effective treatment or prevention method, especially when weighed against effects on bone loss, in MM is needed.

A greater understanding of how obesity contributes to drug resistance in MM is needed, as patients commonly relapse after therapies becomes ineffectual. The development of relapsed/refractory MM is the most common cause of MM death. The treatment regimen for newly-diagnosed MM patients depends on their risk as determined by genetic mutations within the myeloma cells themselves, as well as the patients’ ability to tolerate autologous stem cell transplantation (ASCT), access to care, and other factors. In transplant eligible patients, frontline therapy typically includes bortezomib (or other proteasome inhibitors), lenalidomide (or other IMiDs [immunomodulatory drugs]), and dexamethasone (an anti-inflammatory, anti-myeloma steroid) for 3-4 cycles prior to ASCT. Patients who are not eligible for ASCT often receive similar treatments, but for 8-12 cycles (54). High-risk patients also may receive daratumumab (54), which binds to CD38, a cell surface marker overexpressed in myeloma cells, to recruit immune cells that target and kill MM cells. Maintenance therapy for patients includes lenalidomide, while bortezomib is added to this in the case of high risk patients. Many other therapies, such as melphalan (a chemotherapy) and newer, more targeted therapies are also used for front-line or maintenance therapy, or explored in the context of a clinical trial. How obesity affects the efficacy or long-term outcomes of these treatments, some of which (eg. dexamethasone) can lead to weight gain, remains a challenge in the field of precision medicine. Omega-3 fatty acids have been shown to enhance the killing effects of bortezomib and induce apoptosis in MM cell lines, but *in vivo* and clinical data are lacking (55). Overall, a better understanding of the effects of obesity on efficacy of current therapies could hold the potential to better tailor treatments to individual patients.

## 4 Biologic mechanisms linking obesity with MM

### 4.1 *In vivo* models

As with all cancer research, murine models of MM are a critical tool to understand the pathogenesis of the disease and in the development of novel therapeutic strategies. No model perfectly recapitulates the human tumor/tumor microenvironment, but many exist for MM and the best one(s) based on the hypothesis being tested should be identified. The choice of the system involves weighing the pros and cons of different model attributes, like the requirement of an intact host immune system, the use of human cell lines, and the host microenvironment. The inoculation of the myeloma cells can also be achieved with different methods such as tail vein injection to induce systemic disease, subcutaneous injection to induce solitary plasmacytomas, or intratibial injections to induce lytic bone lesions in the limb inoculated (56).

Many models simply do not show any effect of high fat diet (HFD) or obesity on MM disease progression, for various reasons. For example, many common immunodeficient strains (eg. severe combined immunodeficiency (SCID), non-obese diabetic/severe combined immunodeficiency (NOD/SCID), and NOD/SCIDIL2Rγ (NSG) mice), used for xenograft MM models, seem to be resistant to developing HFD-induced metabolic syndrome, and often do not become obese, due to a lack of adaptive immunity and defective innate immunity (55). In our lab, we found no significant differences in either HFD or weight cycling (high fat and low fat cycled dieting) in terms of survival compared to control diet, in a male SCID-beige MM.1S xenograft model (57).

**Figure 1:**
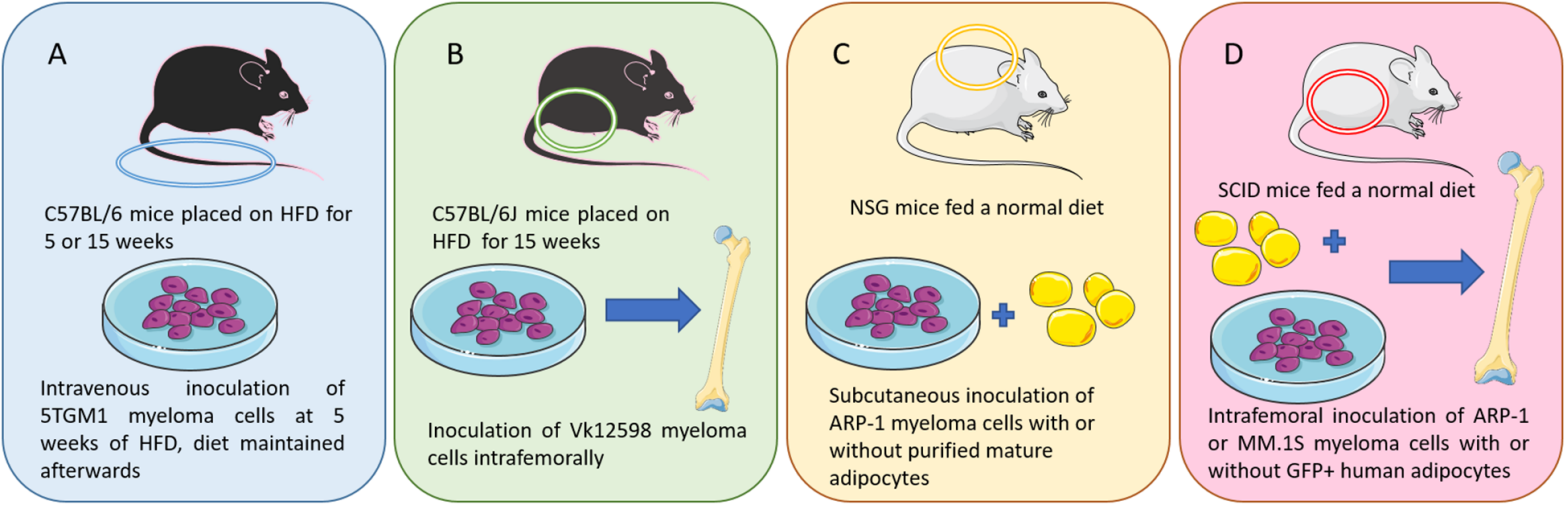
Murine models of multiple myeloma: **(A)** I.V. injection of 5TGM1 MM cells in C57BL/6 mice on a high fat diet (a diet-induced obesity model) (58). **(B)** Intrafemoral injection of Vk12598 MM cells in C57BL/6J mice on a high fat diet (a diet-induced obesity model) (59). **(C)** Subcutaneous inoculation of ARP-1 MM cells and adipocytes in NSG mice (59). **(D)** SCID mouse femurs injected with MM cells (ARP-1 or MM.1S) or co-injected with MM cells and GFP-labeled mature human adipocytes (60). Figure includes modified templates from Servier Medical Art.

However, some models can be used to investigate how obesity contributes to MM. In a 2015 study, Lwin et al., described the first DIO model useful to examine effects of obesity on MM development *in vivo* (58). C57BL/6 mice, which are not normally permissive hosts for 5TGM1 murine myeloma cells, were placed on HFD (42% fat) or control diet (CD) for 5 weeks (**Figure 1A**). A significant increase in body fat and body weight were observed among the HFD mice. The mice were then inoculated with 5TGM1 myeloma cells *via* tail vein and their diet was maintained during the experiment. C57BL/6 mice on HFD developed features of MM including a significant increase in myeloma specific IgG2_bk_ paraprotein and an accumulation of GFP-positive myeloma cells in the BM and spleen, while the mice on CD did not exhibit these characteristics. MicroCT analysis of the tibial trabecular bone volume (TBV) also showed significant bone loss in the myeloma-bearing mice on HFD (58). In this same study, myeloma permissive C57BL/KaLwRij mice were placed on HFD for 5 weeks before inoculation with 5TGM1 cells (tail vein), which resulted in tumor growth and osteolytic bone disease (58). Despite an increase in body fat due to the HFD, no significant differences in serum paraprotein, tumor burden within the BM, or TBV were observed. This indicates that the obese host environment created by HFD may not be directly promoting tumor growth or survival, but may be creating a myeloma-permissive microenvironment. Also in that manuscript, mice with a mutation in the leptin gene (*ob/ob*) resulting in biologically inactive leptin, were used to analyze myeloma development in a genetic model of obesity. 5TGM1 myeloma cells were inoculated into 12-week-old *ob/ob* mice *via* tail vein injection. No increase in serum paraprotein was observed and no GFP positive myeloma cells were detected in the BM or the spleen, perhaps due in part to the fact that leptin itself is a pro-myeloma adipokine and thus pro-tumor effects of obesity may be negated by the anti-tumor effects of leptin removal in these mice (61). This shows that there are distinct differences created by diet-induced obesity and leptin deficiency-induced obesity in terms of effects on tumors (58).

To investigate these differences between the models further, serum concentrations in DKK1 and adiponectin, as well as IL-6 and insulin growth factor (IGF)-1, which are connected to both obesity and myeloma, were measured (58). HFD was found to have no significant impact on DKK1 and total adiponectin concentrations compared to *ob/ob* mice, suggesting that these factors are not responsible for the myeloma-permissive environment generated by HFD. However, diet-induced obesity was found to significantly increase serum IGF-1, while no significant difference in IGF-1 concentration was observed in *ob/ob* mice. This suggests that the increase in IGF-1 is specific to diet-induced obesity and may be contributing to the development of myeloma. IL-6 was not found to be altered in either *ob/ob* mice or HFD mice, but was significantly increased in tumor bearing mice on HFD, suggesting that it came from myeloma cells, or that MM cells stimulated its increase in neighboring cells (58).

Increased myeloma incidence and development under obese conditions was also observed using a second diet-induced obesity (DIO) C57BL/6J mouse model (**Figure 1B**) (59). In this study, mice were injected with murine myeloma Vk12598 cells (which express high levels of *Myc*) into the femurs (intrafemoral). Tumor burden assessed with the serum levels of M-proteins was significantly higher in DIO mice compared with control diet mice. DIO mice also exhibited increased numbers of marrow and spleen-infiltrating CD138+ myeloma cells, as well as enlarged spleens (59). In a third model, within this same publication, Yang et al. used luciferase-labeled ARP-1 human myeloma cells (**Figure 1C**), mixed with purified mature adipocytes obtained from normal BM-derived mesenchymal stromal cells (MSCs) (59). Those were then injected subcutaneously into NOD-scid IL2RG^null^ (NSG) mice fed with irradiated rodent diet. They found that adding human or mouse adipocytes to myeloma cells increased the levels of bioluminescent activity and tumor weights. Similar results were observed with intrafemoral injections of MM.1S or ARP-1 in SCID mice (**Figure 1D**). In addition, whether the murine adipocytes came from normal diet mice or HFD mice differentially affected tumor growth; higher bioluminescent activity was observed in the flanks of the mice injected with adipocytes isolated from HFD vs control diet mice (59).

### 4.2 Bone marrow adipose tissue

**Figure 2:**
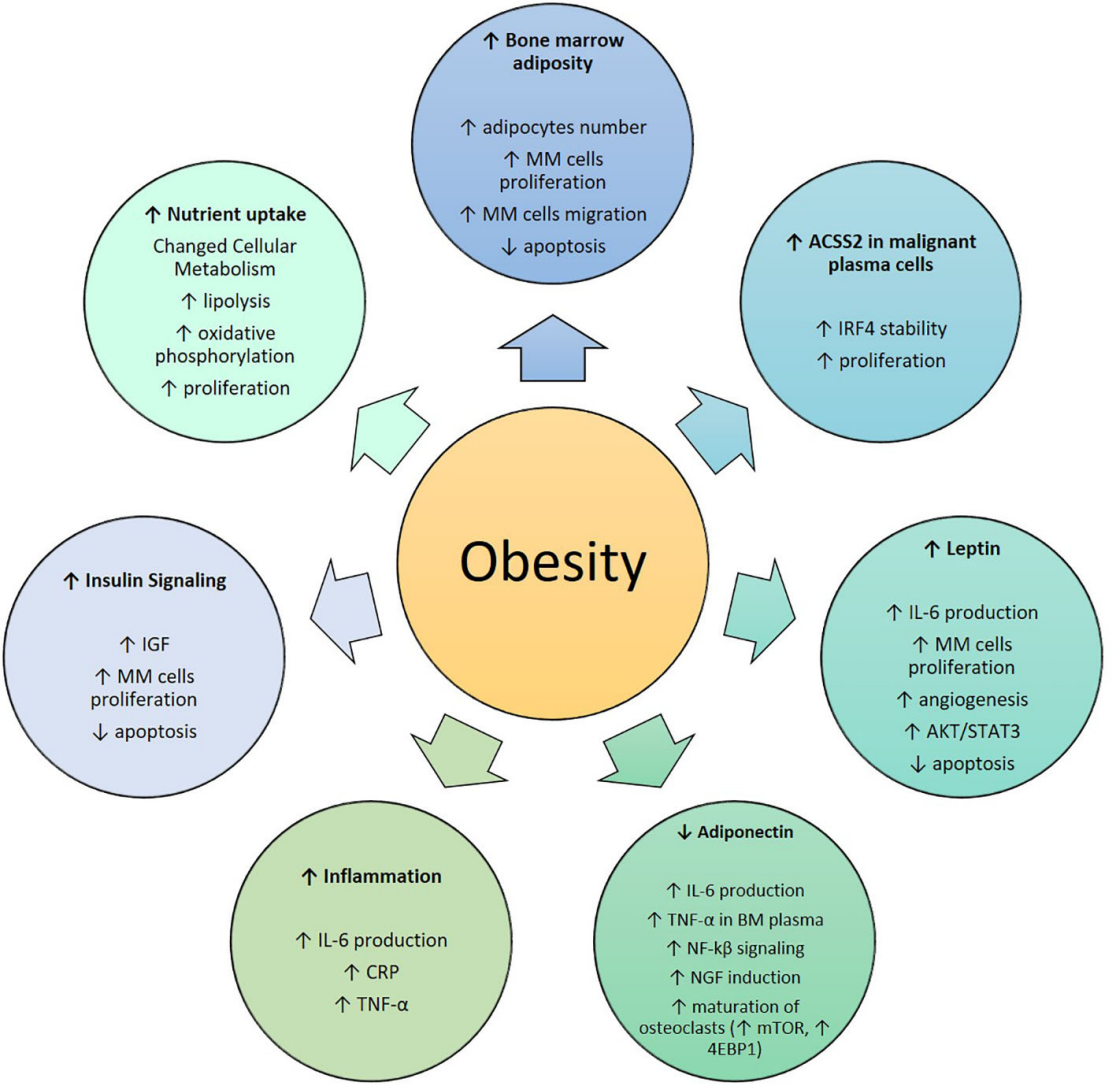
Role of obesity in Multiple Myeloma: MM, Multiple Myeloma; ACSS2, Acetyl-CoA Synthetase 2; IRF4, Interferon Regulatory Factor 4; IL-6, Interleukin-6; AKT/STAT, Protein Kinase B/Signal Transducer and Activator of Transcription; TNF-α, Tumor Necrosis Factor-α; NF-kβ, Nuclear Transcription Factor kβ; NGF, Nerve Growth Factor; mTOR, Mechanistic/Mammalian Target of Rapamycin; 4EBP1, Eukaryotic Translation Initiation Factor 4E-Binding Protein; CRP, C-Reactive Protein.

Obesity is known to cause an increase in BMAT in mice and humans (**Figure 2**), highlighting the potential for increased or altered BMAT to be a major link between obesity and MM (62–64). One of the first studies to investigate a potential link between BM adipocytes and MM cells was from Caers et al., where 5T33MM murine myeloma cells and the human MM5.1 cell line were used (65). In this work, normal and concentrated conditioned medium (CM) from BM fibroblasts, BM adipocytes and peripheral adipocytes were added to 5T33MM cells, and their DNA synthesis was measured. A significant increase in DNA synthesis in MM cells was detected in response to BM adipocyte media compared to the control. Direct cell-cell contact between MM cells and adipocytic cells was also found to increase DNA synthesis significantly in murine 5T33MM cells. Fluorescence activated cell sorting (FACS) analysis of caspase-3 activity also showed that adipocytic cells protect MM cells from apoptosis. The migration assay revealed an enhanced migration of murine MM cells towards concentrated CM of BM adipocytes. Therefore, BM adipocytes affect proliferation, apoptosis and migration of MM cells (65). However, as MM cells invade the BM, BM adipocytes tend to disappear and change (eg. display a SASP, senescence-associated secretory phenotype, or become reprogrammed in other ways to induce bone resorption) during the disease development (66–71). This suggests that a bi-directional relationship exists between adipocytes and MM cells, and that the main role of adipocytes may be in the initial stages of the disease.

A combination of both *in vitro* and *in vivo* methods have been used to test the hypothesis that an increase in adipocyte quantity promotes the progression to MM. In 2016 Trotter et al. (72) were among the first to show that the BM from patients having MM contained more preadipocytes and larger mature adipocytes than normal BM. They was also found that preadipocytes and mature adipocytes secrete many molecules that support the growth of MM cells in the BM and recruit MM cells *via* both stromal cell-derived factor-1-a and monocyte chemotactic protein-1. In addition, CM from mature adipocytes augmented MM growth, and co-culture with preadipocytes resulted in increased MM cell chemotaxis *in vitro*. This supported the importance of adipocytes on MM progression and suggested they represent a specific target in the BM microenvironment (72). In 2019, using the C57BL/KaLwRij murine model of myeloma, BM adiposity was found to be increased in early stage myeloma, while bone marrow adipocytes (BMAds) were localized primarily along the tumor-bone interface at later stages of disease (69). Myeloma cells were found to uptake BMAd-derived lipids *in vitro* and *in vivo*, although lipid uptake was not associated with the ability of BMAds to promote myeloma cell growth and survival (69). The Yang laboratory has shown that adipocytes activate autophagy in myeloma cells, *via* the STAT3 signaling pathway, leading to chemoresistance and reduced apoptosis using eloquent *in vivo* and *in vitro* studies (60). Our work has also found that BMAT induces drug resistance in MM cells (70). New, tissue engineered 3D models of BMAT have also shown that BM adipocytes shrink with MM cell co-culture; these models may be useful to recapitulate tumor-host interactions in more physiologically relevant conditions than can be achieved with 2D cultures, but with more control than possible *in vivo* (73, 74).

MM cells use mitochondrial-based metabolism as well as glycolysis in the BM (75). It was recently reported that intercellular mitochondrial transfer from neighboring nonmalignant BM stromal cells to MM cells *via* tumor-derived tunneling nanotubes (TNT) can change cellular reliance on oxidative phosphorylation, and hence BMAds could not only provide fatty acids, adipokines, and other fuel, but also, potentially mitochondria, which could affect MM cell survival and growth (75). This is an area open for interrogation.

### 4.3 Lipid mediators and metabolism-related enzymes

The roles of lipids and other metabolites in patient serum is also under investigation as a mechanism by which obesity may affect MM. For example, PI resistance may be partially mediated by oxidized LDL, a central mediator of atherosclerosis that is elevated in obesity (76). Oxidized LDL suppressed the boronic-acid based PIs (bortezomib and ixazomib) mediated killing of human MM cell lines through both proteasome inhibition and pro-apoptotic signaling (76). This implies that patients with metabolic syndrome (obesity, insulin resistance, dyslipidemia, and hypertension) could see deepened clinical response to these PIs when supplemented with cholesterol-lowering therapy. However, this is not supported by a 2008 study that found total cholesterol (TC), low-density lipoprotein cholesterol (LDL-C), and high-density lipoprotein cholesterol (HDL-C) in patients with MM were significantly lower than the healthy controls (77). Similarly, a new 6-prognostic factor model was constructed based on Lasso regression to assess the following serum lipids in MM patients: triglyceride (TG), low-density lipoprotein (LDL), high-density lipoprotein (HDL), lactate dehydrogenase (LDH), Apolipoprotein B (Apo B) and Apo B/Apolipoprotein A1 (Apo A1) ratio (78). Together, these created a prognostic model, identified through univariate and multivariate Cox analysis, which exhibited better accuracy than International Staging System (ISS) and Durie and Salmon (DS) stage for 5- and 10-year OS. Data from the model support ApoB and the ApoB/ApoA1 ratio as being associated with shorter OS, but total cholesterol and HDL-C levels with being associated with longer OS (78). Still, the direction of the causal relationships between tumor development and changes in serum lipid levels is not known from the current data, and compensation mechanisms may be at work. Interestingly, *in vitro*, accumulation of lipids in MM cells was induced by PI treatment, and lipid-lowering drugs combined with a PI exerted a synergistic killing effect on myeloma cells (79).

Differences in circulating lipids or other metabolites may also affect tumor cell metabolism, and lipid metabolism within MM cells, in normal or high BMI patients, is of great interest. In a study analyzing myeloma cells isolated from obese patients (59), the involvement of the acetyl coenzyme A (Acetyl-CoA) metabolic process in obesity-associated myeloma growth was observed. The production of Acetyl-CoA from acetate is primarily dependent on Acetyl-CoA synthetase 2 (ACSS2), the enzyme which catalyzes the synthesis of Acetyl-CoA from short chain fatty acids. It was found that *ACSS2* was highly expressed in malignant plasma cells from MM patients. This expression was even higher in obese patients, and a correlation between *ACSS2* expression and patient BMI was established. In subsequent experiments, adipocyte-secreted angiotensin II was identified as the direct cause of this increased expression of *ACSS2*. ACSS2 interacts with the oncoprotein interferon regulatory factor 4 (IRF4), and enhances IRF4 stability and IRF4-mediated gene transcription through activation of acetylation. IRF4 plays a main role in B-lymphocyte development and acts as a transcription factor, regulating the expression of genes supporting myeloma growth and survival. As the use of an ACSS2 inhibitor reduced myeloma growth both *in vitro* and in a diet-induced obese mouse model, the critical role of ACSS2 in MM was verified (59). Another fatty acid metabolism gene, fatty acid elongase 6 (ELOVL6), was recently shown to regulate PI resistance in MM (80). We have found that the ACSL and FABP (fatty acid binding proteins) families of are other, novel potential targets in MM (81–83).

Bioactive lipid mediators may also be new targets in MM. Recent work from the Lynch lab has shown that knockdown of the acid ceramidase enzyme, ASAH1, which typically breaks down lipids of the ceramide class, led to reduced conversion of ceramide to sphingosine 1-phosphate (S1P) and decreased expression or activity of the anti-apoptotic proteins (MCL-1, BCL2 and BCL-xL) along with increases in pro-apoptotic proteins (BIM and NOXA) (84). Notably, ASAH1 knockdown also significantly sensitized the MM cells to PI treatment (84). In further support of this, S1P activation has been shown to contribute to proliferation and survival of MM cells (85). More information on targeting lipid metabolism in MM cells can be found here (86), and a review of metabolic changes in the BM microenvironment to relate to MM progression can be found here (87).

### 4.4 Adipokines

In addition to angiotensin II, as mentioned above, adipose tissue expresses and secretes multiple bioactive peptides called adipokines that act both on the local and systemic level. Adipokines regulate a broad range of activities including angiogenesis, oxidation, and cellular signaling. During metabolic diseases, including obesity, increases in adipose tissue result in subsequent increased production and secretion of many adipokines, with adiponectin a notable exception (88, 89). Leptin, an important regulator of caloric intake and metabolic function, is elevated in obese humans (89). It has been shown to be highly correlated with BMI in mice, and weight loss due to food restriction causes a decrease in the leptin levels (90). Caers and colleagues confirmed that adipocytes were the only cells within the MM microenvironment to secrete leptin, by RT-PCR and ELISA (65). Leptin also appears have proliferative and anti-apoptotic effects in myeloma cells (61, 91). Leptin has been shown to be responsible for reducing the anti-tumor effects of chemotherapy *via* activation of AKT and STAT3 pathways, as well as upregulating Bcl-2 expression and inhibiting caspase-3 activation in MM cells *in vitro* (61).

Adiponectin is one of most highly expressed substances in adipocytes, and was found to be decreased in obese patients (92). In a case-control study, the role of serum adiponectin in MM was investigated by analyzing blood samples collected from 73 patients with histologically confirmed MM and 73 non-tumor bearing controls. Lower serum adiponectin levels were associated with higher risk of MM by bivariate analysis (93). While investigating whether circulating levels of total adiponectin and high molecular weight (HMW) adiponectin were associated with MM risk among 174 MM patients, an inverse relationship between total and HMW adiponectin levels and subsequent risk of MM was shown (94).

Much work from the Edwards lab has supported the anti-myeloma role of adiponectin in MM (95). Recently, from the Edwards lab, myeloma cells were found to downregulate adiponectin specifically in BMAds but not in white adipocytes (69). They found the ability of myeloma cells to downregulate adiponectin was dependent in part on TNF-α which was significantly correlated with tumor burden in the BM plasma of myeloma-bearing mice. To investigate how TNF-α downregulates BMAd-derived adiponectin, transwell co-culture of myeloma cells with BMAds was used, and it revealed an increase in activation of JNK, p38MAPK and ERK1/2, pathways that are implicated in TNF-α mediated suppression of adiponectin in WAT (69). Mouse recombinant TNF-α induced a significant decrease in *Adipoq* expression and in secreted adiponectin in ST2-derived mouse BMAds. Conversely, the addition of a neutralizing antibody to TNF-α in myeloma/BMAd co-cultures blocked the suppression of adiponectin and prevented the reduction in BMAd number. This work demonstrated that myeloma cells down-regulate adiponectin in BMAds *via* TNF-α (69). Adiponectin signaling may also help reduce bone pain, through a TNF-α-NF-kβ-adiponectin axis that regulated nerve growth factor (NGF) and pain signaling in MM (96). A recent study also found a correlation between adiponectin and markers of MM bone disease and further investigated a potential mechanism of action of adiponectin on the differentiation and maturation of osteoclasts in MM (97). Flow cytometry was used to detect the expression of adiponectin receptor 1 (AdipoR1) and the phosphorylation of the mechanistic target of rapamycin kinase (mTOR) and eukaryotic translation initiation factor 4E-binding protein (4EBP1). It was found that adiponectin inhibits the differentiation and maturation of osteoclasts by increasing the expression of AdipoR1 and reducing the phosphorylation levels of mTOR and 4EBP1 in patients with MM (97).

Data on the role of resistin do not clearly define the role of this adipokine in MM. In 2017, Pang et al. showed that resistin contributes to muti-drug resistance in MM cells by inhibiting cell death and upregulating ATP-binding cassette transporter expression (98). However, lower serum resistin levels correlated with higher risk of MM in one clinical study, adjusted for BMI and other factors, but it is possible that resistin levels were decreased in a compensatory/response mechanism in this case (93). Thus, more research into the roles of resistin, and other adipokines, in MM would be valuable.

### 4.5 Hormones and inflammatory cytokines

Obesity is often characterized as a hyperinsulinemic state. Insulin and IGF-I are potent growth and survival factor for MM cells (96). MM cell lines express IGF-I, IGF-II, and insulin receptors; insulin and IGFs can protect MM cells from dexamethasone-induced apoptosis and thus play a role in maintenance of the malignant clone (99). Work from the Rudikoff laboratory studied the IGF-I signaling cascade in 8 MM cell line; in addition to inhibiting apoptosis, IGF-I was found to activate the MAPK pathway, resulting in proliferation. Moreover, *in vivo* administration of IGF-I in SCID mice inoculated with the OPM-2 cell line led to a tumor growth rate twice as high as in the controls (100, 101). Inhibition of the IGF-I pathway was not found to change the proliferative effect of IL-6, and IL-6 and IGF-I were found to activate different downstream signaling molecules. Thus, IGF-I was found to act as a survival and proliferation factor for MM cells by stimulating an IL-6 independent signaling cascade (102).

Free serum sex hormones, eg. estrogen and testosterone, are is increased in obesity in part due to a decrease in the sex hormone-binding globulin (SHBG) (103, 104). These hormones interact with numerous pathways throughout the body that likely affect MM. For example, estrogens have been shown to promote MM by enhancing the immunosuppressive activity of myeloid-derived suppressor cells (MDSCs) (105). The estrogen-responsive gene microtubule-associated serine/threonine kinase family member 4 (MAST4) was also recently found to be a critical factor in MM-induced bone disease (106). Still, the relationship between estrogens and MM is not completely clear, because another study found that activation of estrogen receptors, which are highly expressed by MM cells, blocked interleukin-6-inducible cell growth of human MM cells (107).

The state of chronic inflammation associated with obesity is related to high levels of inflammatory cytokines such as IL-6, C-reactive protein (CRP), and TNF-α. IL-6 has been long known to be involved in inhibition of MM cell apoptosis and promotion of their survival (108). Interactions of IL-6 with adhesion molecules, cytokines, such as transforming growth factor beta-1, tumor suppressor genes and oncogenes, lead to the survival of malignant plasma cells (109). In addition, IL-6 is also suggested to cause drug resistance using epigenetic modulation proteins. IL-6 also enhances DNA methyltransferase-1, and thus promotes methylation and deactivation of p53, enabling MM cells to avoid apoptosis (110). CRP is a polypeptide protein secreted by hepatocytes in response to IL-6 and represents an indicator of IL-6 production. TNF-α induces expression of adhesion molecules on MM cell lines and on BM stromal cells leading to increased binding of MM cells to BM stromal cells and increased IL-6 secretion (99). The direct and indirect roles of other immunomodulatory interleukins, such as IL-10 (111), and the IL-12 family cytokines (112, 113), the gut microbiome (known to change based on diets and obesity) and adipocyte-derived chemoattractants such as MCP1 (also known to change in obesity (112),) are multifactorial and would benefit from further investigation (114).

### 4.6 Nutrient uptake

Metabolic transformations are one main aspect of cancer, and targeting these transformations is one critical lead for cancer therapy. Cancer metabolism and phenotype rely on cell-intrinsic factors, such as metabolite availability in the tumor microenvironment (TME). A large range of cell types contribute to the metabolite composition of the TME, and are involved in tumor cell metabolism, interactions between cancer and non-cancerous cells, and whole body metabolic homeostasis (115).

In work from the Raje laboratory using murine MM cells and BM adipocyte coculture assays, MM-induced lipolysis in adipocytes *via* activation of the lipolysis pathway was investigated (71). In this work, MM cells were shown to induce lipolysis in adipocytes using a glycerol secretion assay. The observation of an upregulation of the genes involved in fatty acid (FA) lipolysis, FA synthesis, and FA desaturation lead to the conclusion that an altered FA metabolism is induced in MM cells (71). In this study, murine 5TGM1 and human-derived OPM2 MM cells were exposed to fluorescently labeled FA and analyzed. Results indicated that the cellular machinery for FA transport are present in MM cells, and lipolysis-induced free fatty acids (FFAs) are transferred intracellularly into the MM cells, potentially altering FA metabolism. Upregulation of fatty acid transporters 1 and 4 on MM cells mediated the uptake of secreted FFAs by adjacent MM cells. The effect of FFAs on MM cells was studied by peritumoral delivery of arachidonic acid on a plasmacytoma MM.1S model in SCID mice. This experiment revealed an increased proliferation at lower concentrations and the induction of lipotoxicity, *via* ferroptosis, at higher concentrations of FFAs. The authors rightly concluded that the prevention of FFA uptake by MM cells could represent a potential target for myeloma therapeutics (71). A deeper understanding of the metabolic flexibility and requirements of tumor cells, and downstream tumor cell changes resulting from different metabolic pathways, would enable novel therapy development in MM and many other cancers.

### 4.7 The immune system

Currently, no studies have specifically examined a three-way link between obesity, the immune system, and myeloma progression, despite the fact that all of the above biological effects or consequences of obesity also have effects on immune cells. However, different pairs of this triad have been studied in depth in MM as we have described above (obesity and MM) and as others have [immune cells and MM (116)]. Fortunately, there are potentially relevant findings in other malignancies that may translate to MM. Here we discuss some of the most recent studies investigating effects of obesity on T cells and macrophages in the TME, which represents a promising direction of research. To better understand the impact of obesity on MM and design novel interventions, the roles of other immune cells should also be investigated, especially now that immunotherapies are becoming so prevalent in MM treatment clinically.

#### 4.7.1 HFD induced obesity impairs CD8+ T cell function in the murine TME

One main feature of tumor cell metabolism is increased nutrient consumption to meet energetic, anabolic and pro-survival demands. As activated T cells are highly proliferative and rely on metabolic pathways to function, tumor cell metabolism and anti-tumor immunity are tightly related. In 2020, Ringel et al. investigated how obesity shifts the metabolic landscape of the tumor microenvironment to inhibit T cell function and promote tumor growth (117), and though they did not use myeloma cells in this work, the possibility of their findings to apply to MM as well remains.

In the Ringel et al. work, to model human obesity in mice, C57BL/6J mice were put on either CD or HFD at 5 weeks of age, and for 8-10 weeks. They were injected with syngeneic MC38 colorectal adenocarcinoma cells, which grew more quickly in the HFD mice than in the CD mice. Flow cytometry was used to profile tumor-infiltrating immune cell populations 10-14 days after inoculation. HFD was found to reduce the number and functionality of intratumoral CD8+ T cells (117). Single-cell profiling revealed that immune cells in the TME go through metabolic changes in response to HFD, and the differences are distinctive in the T cells. Although CD8+ T cells are found within HFD tumors, it seems that HFD changes metabolic niche interactions within tumors and impacts local T cell infiltration patterns. These metabolic adaptations differ from the ones affecting tumor cells, which lead to altered fatty acid partitioning in HFD tumors, impairing CD8+ T cell infiltration and function. HFD MC38 tumor cells rewire metabolism to increase fatty acid uptake and oxidation. Indeed, HFD decreased expression of prolyl hydroxylase 3 (*PHD3*), a critical metabolic regulator, in MC38 cells. Restoring *PHD3* expression in tumor cells was found to be sufficient to alter nutrient availability in the TME. Investigating the effect of *PHD3* overexpression on tumor growth *in vivo*, data showed that maintaining high PHD3 expression in MC38 tumor cells improved the anti-tumor T cell response in HFD mice. Analysis of other human cancers revealed similar changes in CD8+ T cell markers, suggesting interventions that exploit metabolism may improve cancer immunotherapy (117).

The effects of obesity on CD8+ T cells were also investigated by Dyck et al. in mouse models and patients with endometrial cancer (118). It was also found that obesity enhances tumor growth and reduces CD8 T cell infiltration, proliferation, and function in the tumor. Suppression of CD8+ T cell infiltration in obesity was associated with a decrease in chemokine production such as IFN-γ. Tumor-resident CD8+ T cells were also found to be functionally suppressed in obese mice, due to a suppression of amino acid metabolism. In fact, CD8+ T cell activation requires the activity of the amino acid transporters SLC7A5, and their activity in MC38 tumors in obese mice was found to be significantly reduced. Glutamine levels were also decreased in mice on HFD, and as it is essential for CD8+ T cell function and is a substrate for SLC7A5, low levels of glutamine could impair CD8+ T cell function in obesity. The immune checkpoint PD-1, which is frequently up-regulated on dysfunctional T cells in tumors, was also found to be more highly expressed in MC38 tumors. Immunotherapy using anti-PD-1 partially restored CD8 metabolism and anti-tumor immunity (118).

#### 4.7.2 Macrophages and tumor cells

Tumor-associated macrophages (TAMs) possess distinct phenotypes. While M1 macrophages have a pro-inflammatory (anti-tumoral) function, M2 macrophages have an anti-inflammatory (pro-tumoral) function. Studies have shown that the polarization of TAMs towards an M1 or M2 phenotype is driven by environmental factors such as cytokines, chemokines and other soluble factors secreted by the neighboring cells. Polarization towards the M2 state has been found to be correlated with a lack in p53 (115). The discoveries regarding the metabolic profile of macrophages represent an interesting therapeutic target: promoting a switch to an M1, anti-tumoral phenotype.

Like T cells, TAMs also compete with their neighboring cells for glucose. Glycolytic activity in TAMs has mainly been associated with tumor regression (115). Moreover, hypoxic TAMs have increased expression of the mTOR negative regulator REDD1 and consequently display decreased glycolysis. Lactate produced by tumor cells also has a critical function in signaling and TAM polarization as it induces a pro-tumoral M2 phenotype (115). Glutamine metabolism in TAMs has also been linked to a pro-tumoral phenotype (115). Monocytes have also recently been shown to respond to HFD in mice, in work from the Klip laboratory: this group found that short-term HFD changed BM cellularity, resulting in local adipocyte whitening, driving a gradual increase and activation of invasive Ly6C^high^ monocytes (119). These monocyte changes preceded a rise in adipose tissue macrophages during HFD in the mice. Moreover, skewing of the BM towards a preponderance of Ly6C^high^ monocytes was preceded by monocyte metabolic reprogramming towards glycolysis, reduced oxidative potential and increased mitochondrial fission. Thus, obesity, or general metabolic state, likely also affects tumor progression through influences on monocyte or macrophage metabolism.

In a recent study by Micallef et al. the C1q/TNF-related protein family member C1qtnf3 was identified as one of the most upregulated genes responsible for secreted proteins in tumor-associated adipose tissue, especially in diet-induced obese mice (120). Macrophage accumulation in tumor-associated inguinal adipose tissue was found to be inhibited by the administration of C1QTNF3 neutralizing antibodies, but tumor growth was unaffected. Moreover, C1QTNF3 treatment of M2 macrophages stimulated the ERK and Akt pathway, increasing the polarization towards the M1 state. These results suggest that macrophages could be recruited to adipose tissue with increased C1QTNF3 production (120). In sum, aiming to redirect a polarization of TAMs towards an M1 phenotype by affecting metabolism, or other signaling pathways, represents a promising therapeutic target.

## 5 Discussion

As evidenced by the scant publications available in the myeloma field, modeling the contributions of obesity to MM progression in mice has been difficult (57). These challenges are compounded by the fact that many myeloma studies utilize human MM cells, which require the use of immunocompromised (eg. NOD/SCID, SCID-beige, or nude) mice, which do not exhibit robust responses to DIO regimens, and are lacking a full immune cell repertoire. Indeed, a thorough investigation of changes in tumor cells and BM immune cell milieu induced by obesity that support MM disease progression has not been executed. Most studies of obesity on MM rely on direct injection of mouse tumor cells into the bones (59) of mice on a HFD, due to the unpredictability of mouse tumor cell engraftment when injected systemically, and often result in growth in locations other than the bone.

The Vk*MYC mouse myeloma cell line Vk12598, which grows in C57BL/6 mice, does induce osteolysis when injected systemically into mice (121), which is even more pronounced when the cells are injected intrafemorally (34). So far, as we described above, this model has only shown a response to DIO when the Vk12598 cells are injected intrafemorally, which is useful but does not recapitulate the series of steps MM cells take in disease progression (ie. extravsation, survival through the circulation, intravasation, homing to the bone marrow) (59). However, murine eGFP+/5TGM1 cells, originally derived from the C57BL/KaLwRij mice (122), can spread to the bone from circulation and cause extensive osteolysis, as we have shown (123). However, they can also spread to other locations, such as the spleen, which poorly models MM clinically (59). Still, these cells have proven useful in a MM DIO model using IV injection (58, 59), and hopefully, using this or a similar immunocompentent, DIO model, the mechanisms of obesity’s effects on MM can be better elucidated. We now know obesity alters the composition of the BM (124), as well as the genes expression profiles of the cells within the niche (125). Obesity is correlated with an increase in BMAds in mice (13) and humans (126), which are known contributors to systemic adipokines such as leptin (pro-myeloma) (94) and adiponectin (anti-myeloma) (95), however the relationship between MM and BMAds has only begun to be investigated. Recent studies by our lab and others have demonstrated significant effects of MM cells on BMAds including decreased lipid content (66, 68–70), increased expression of inflammatory cytokines (66, 70), and decreased adiponectin (69, 70), with myriad implications for direct interactions with MM cells, as well as the vicious cycle of MM-induced bone disease. Whether these myeloma-associated adipocytes are involved in homing, engraftment, or *in vivo* drug resistance should be further investigated in animal models going forward, since we and others have shown that adipocytes promote drug resistance in myeloma cells *in vitro* (60, 70, 98).

Obesity, like aging (127), is now being linked to senescence in the BM microenvironment (125) and cellular senescence and cancer susceptibility are correlated in a number of conditions (128), begging the question of whether aging and obesity-induced senescence in the marrow might actually drive myelomagenesis. Recent data from the Weivoda laboratory support this idea, based on clinical data and mouse models showing that MGUS and smoldering MM plasma cells are in a senescent-like state, and data that targeting senescence in these early MM diseases can reduce plasma cell numbers in the MGUS model (129). Moreover, *in vitro* studies suggest that myeloma cells might induce senescence in the marrow in mesenchymal stromal cells (14, 20, 21) and BMAds (13), however the relationship between senescent cells and myeloma is still not completely understood. Promising preclinical studies in both aged (127) and obese (130) mice, and preliminary reports from a clinical trial (131), demonstrate efficacy of senolytic therapies to target and clear senescent cells in obesity models and aging. Future studies should investigate a role for senolytics in combination with traditional myeloma chemotherapies and treatments, particularly since MM therapies often induce senescence.

## 6 Conclusion

Obesity has been implicated in myeloma risk and transformation from MGUS to MM, as well as MM mortality. Direct mechanisms tying obesity-driven increases in adipose tissue to tumor cell proliferation and survival through secretion of cytokines such as leptin and IL-6, or to myeloma cell drug resistance are being unveiled. The three-way relationship between myeloma cells, adipocytes, and the immune system, and the potential for metabolic- or senescence-focused therapies should be examined going forward. Preclinical studies, including the establishment of a reliable, bone-homing, immunocompetent DIO model are critical to understanding the complex molecular mechanisms at play in the obesity-myeloma relationship.

## Author contributions

CM-M and RI researched and wrote the article. HF and MR researched, wrote, reviewed and edited the article. All identified the theme of the review. All authors contributed to the article and approved the submitted version.
